# Evaluation of Salicylic Acid and Methyl Jasmonate as Elicitors in *Phyllanthus acuminatus* Hairy Roots by Non-Targeted Analysis Using High-Resolution Mass Spectrometry

**DOI:** 10.3390/molecules29010080

**Published:** 2023-12-22

**Authors:** Katherine Benavides, Andrés Sánchez-Kopper, Karol Jiménez-Quesada, Raquel Perez, Giovanni Garro-Monge

**Affiliations:** 1 Centro de Investigación en Biotecnología, Escuela de Biología, Instituto Tecnológico de Costa Rica, Cartago P.O. Box 159-7050, Costa Ricakjimenez@itcr.ac.cr (K.J.-Q.);; 2 Centro de Investigación y de Servicios Químicos y Microbiológicos, Escuela de Química, Instituto Tecnológico de Costa Rica, Cartago P.O. Box 159-7050, Costa Rica

**Keywords:** *Phyllanthus acuminatus*, hairy roots, elicitation, phenylpropanoids, terpenoids, phenols, flavonoids, methyl jasmonate, salicylic acid

## Abstract

*Phyllanthus acuminatus* has been studied for its vast medical and industrial potential. Phytochemical investigations reveal that the genus is a rich source of lignans, flavonoids, phenolics, terpenoids, and other metabolites. However, the phytochemical profile elucidation of this species still needs further research. The use of eliciting compounds such as salicylic acid and methyl jasmonate has managed to increase the production of secondary metabolites in plant cell cultures. Hairy roots of *Phyllanthus acuminatus* were produced in 250 mL flasks with a 16 h light/8 h darkness photoperiod under diffused light with a culture time of four weeks. The elicitors salicylic acid and methyl jasmonate were tested in 50 μM and 200 μM concentrations. Non-targeted analysis was done for the different treatments using HR-MS. Identified metabolites were grouped in phenylpropanoids, phenols, and mucic acids, and statistical analysis of relative concentrations was achieved. A significant change in phenols’ relative concentrations appeared in the elicitations with salicylic acid. Because of the elicitation treatment, specific compounds increased their concentrations, some of which have known pharmacological effects and are used in treating chronic diseases. The best elicitation treatment was salicylic acid 50 μM as it increased by more than 100% the general content of phenols and phenylpropanoid derivates and triplicates the concentration of mucic acid derivates in treated hairy root extracts. The application of non-targeted analysis showed interesting changes in phytochemical concentration due to elicitation in *Phyllanthus acuminatus* hairy roots.

## 1. Introduction

*Phyllanthus acuminatus* is a species of the Phyllanthaceae family. *Phyllanthus acuminatus* corresponds to a small tree that can grow between 1.5 and 7 m in height, with light green glabrous leaves, dioecious flowers organized in fascicle-type inflorescences, a brown stem that secretes whitish latex, and purple fruits. This species is distributed throughout the Americas from northern Mexico to northern Argentina. Due to its multiple biological activities, *P. acuminatus* extracts have been tested for pest control, food preservation, and medicinal uses such as treatment of urinary tract disorders, liver diseases, intestinal infections, jaundice, diabetes, and dermatosis, among other ailments [1,2,3,4].

*Phyllanthus acuminatus* has recently been studied for its vast medical and industrial potential. Pharmacological activities for *Phyllanthus acuminatus* extracts and specific metabolites include anticancer, hepatoprotective, antidiabetic, antimicrobial, and cardioprotective effects [5]. Phytochemical investigations reveal that the plant is a rich source of lignans, tannins, flavonoids, phenolics, terpenoids, and other secondary metabolites.

Bioactive compounds of *Phyllanthus acuminatus* are found mainly in its roots. Some of the interesting substances found in roots are secondary metabolites; their concentrations within the plant are low and fluctuate due to geographic, seasonal, and environmental variations, making field plant extracts economically impractical since large land extensions would be needed to recover a reasonable amount of biomass. Thus, the cultivation of in vitro organs through genetic engineering is a feasible alternative for producing phytochemicals of interest [6].

Hairy root culture has gained preference since it has the advantages of being genetically and biochemically stable, having enzymatic potential similar to the mother plant, and being relatively cheap to cultivate [7,8,9]. Hairy roots consist of a tumor tissue growth caused by infection of roots with *Agrobacterium rhizogenes,* proven to be a relevant alternative for plant secondary metabolite production because they have fast growing rates and are able to grow without phytohormones, displaying higher stability than undifferentiated cells [9].

**Figure 1:**
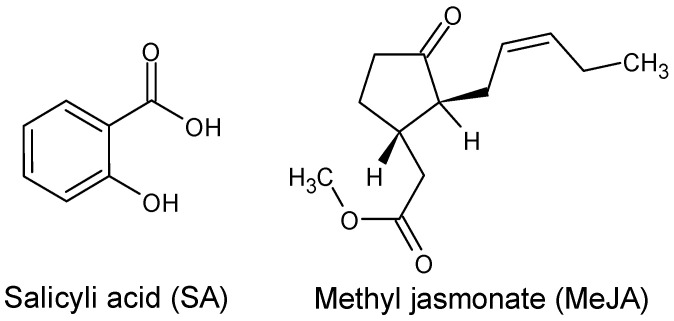
Elicitors molecular structures.

Plant tissue and cell culture are beneficial alternatives for obtaining metabolites of interest because their production is independent of external factors, and nutrients are provided. In addition, the conditions can be controlled to achieve higher yields, genetic stability, and better crop quality. In vitro culture of *Phyllanthus* sp. is feasible since its cultivation does not require complex nutrients, growth medium, or other special conditions [6,7]. Also, hairy root extracts of *Phyllanthus amarus* were shown to induce apoptotic cell death in human breast cancer cells, indicating that the generated plant material possesses interesting bioactive metabolites [8]. Tissue culture also allows a tissue source to be maintained for testing throughout the studies, conserved in the short and medium terms [10]. On many species, including *Phyllanthus pulcher*, eliciting compounds such as salicylic acid (SA) or methyl jasmonate (MeJA) (Figure 1) has increased the production of secondary metabolites. Jasmonic acid (JA) and MeJA are the compounds most commonly studied and widely used as excitons for promoting the accumulation of secondary metabolites in horticultural products, improving their nutritional quality [6]. MeJA has been proven to alter the levels of various primary metabolites, including plant sugars, organic acids, and amino acids [11,12]. Elicitors have received wide attention for enhancing the productivity of plant cells and organs and for being effective in improving secondary metabolite production in cell and organ cultures [13].

Non-targeted screening in plant extracts using MS^E^ data-independent acquisition has been implemented successfully in many studies, improving the accuracy in compound identification since it can simultaneously acquire low- and high-energy spectra of each detected feature, providing information on the precursor and fragment ions [14,15,16].

Our study reports the use of elicitors on *Phyllanthus acuminatus* hairy roots cultures. The effects of SA and MeJA were evaluated for secondary metabolite elicitation. The putative identification of more than 150 compounds present in *Phyllanthus acuminatus* was assigned in the elicitation experiments by means of HR-MS (high-resolution mass spectrometry), and their normalized abundances were compared for the different treatments. Grouping identified compounds by class as phenols, phenylpropanoid derivates, and terpenoids allows us to overview the metabolic profile chances due to elicitors. Principal observed effects were the increment of phenols, mucic acid derivates, phenylpropanoids, and flavonoids by means of SA treatment and the increase in concentration for specific phyllanthoides using MeJA as elicitor. These results demonstrate the feasibility of obtaining interesting bioactive compounds from *Phyllanthus acuminatus* plant tissue cultures as an alternative for improving their industrial accessibility.

## 2. Results and Discussion

The *P. acuminatus* extracts are credited with great antitumor, anticancer, antimalarial, antihepatotoxic, and antimicrobial capacity [7].

Cultivating roots and shoots is widely recommended to increase the production of secondary metabolites since they characteristically have a metabolic pattern remarkably similar to that of the plant’s organs and may be genetically transformed [10,17].

To optimize cell culture conditions, the addition of biosynthetic precursors and application elicitors has been shown to be a practical approach to increasing secondary metabolite production [18]. Plants treated with biotic and abiotic elicitors may develop defense responses similar to pathogen infection effects or environmental stimuli, which can be one of the most effective ways to improve the yield of phytochemicals [19]. Elicitation of secondary metabolites was reported to be the most successful method in cell and organ cultures [20,21,22]. The possibility of enhancing the accumulation of secondary products by adding elicitors to the medium has been extensively studied in cell cultures [13,23].

This study looked at the phytochemical profile of *Phyllanthus acuminatus* hairy roots cultures. The results included identifying the compounds in the cell plant cultures and the effect the elicitation treatments had on these features, followed by the individualized effect each treatment had on the elicitation of specific compounds. Sample separation using liquid chromatography and high-resolution mass spectrometry independent data acquisition experiments made it possible to acquire information on many phytochemicals in the sample extracts. The measured compounds were characterized then by the exact mass of their precursor ions, retention times, chromatographic area, and associated fragments. Assigning putative identifications to the compounds using structure-based databases and comparing acquired spectra with theoretically generated fragments was possible.

Grouping the compounds and summarizing their total standardized peak areas allowed tendency visualization of the compound groups due to the different elicitation treatments applied to the biological material.

Positive and negative ionization data gave different information about compounds because of their differences in adduct formation. Each ionization mode was grouped separately for the overview, and compound intensities were also evaluated to observe specific elicitation.

The putative identification assigned to the measured compounds and their characterization is described in Tables S1–S4. Confirmation of the putative identification was considered when the compound presented a mass error better than 5 ppm; the isotopic distribution match was better than 90%, and the assignation of at least three theoretical fragments.

An initial phytochemical exploration of *Phyllanthus* species reported the occurrence of terpenoids, alkaloids, glycosides, flavonoids, tannins, and saponins [11,12]. Phenolic compounds, especially tannins, are the major constituents of *Phyllanthus* plants. This is congruent with what was observed for the studied hairy root extracts in which phenols constituted 25% of the identified compounds. In the current phytochemical profile, we found the main constituents of *Phyllanthus acuminatus* to be very similar to other genre species. More than 100 phenolic constituents with diverse biological activities were comprehensively identified in the fruits of *P. emblica* L. using HPLC-MS [24]. Notably, different parts of *Phyllanthus* plants have different isomers of the same compounds.

**Figure 2:**
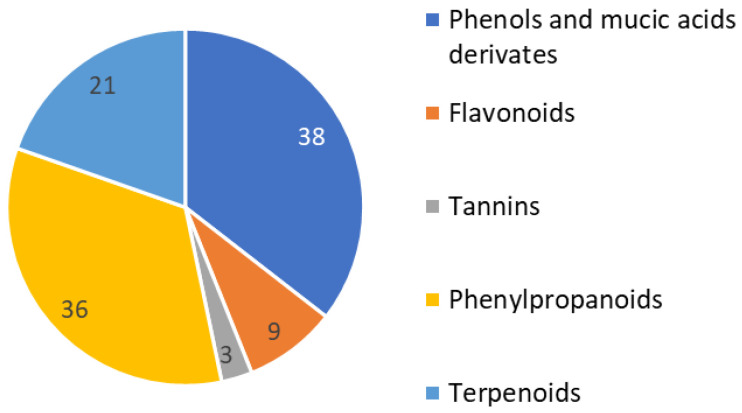
Identified compounds in *P. acuminatus* hairy root extracts grouped by compound type.

For the current evaluation of *Phyllanthus acuminatus*, 28% of the constituents were identified as phenylpropanoids. Almost 81 compounds have been isolated from *Phyllanthus* spp. During the time period 2016–2018, most were phenylpropanoids, triterpenoids, diterpenoids, and flavonoids [25] (Figure 2).

High-resolution mass spectrometry has shown to be a versatile tool for evaluating complex samples. Before an absolute quantitative evaluation of biological extracts, using a non-targeted approach with relative compound quantification is highly informative and brings a first overview of metabolism behavior.

Considering the available phytochemical information for evaluating *P. acuminatus* hairy roots extracts from different elicitation treatments and their putative identifications, it was possible to use relative quantification to compare the effects in the production of compounds with interesting biological activities.

### 2.1. Elicitation Effect Overview by Compound Type Using MeJA and SA as Elicitors

*Phyllanthus acuminatus* hairy root cultures were elicited using MeJA and SA. From the non-targeted measurements of hairy root extracts, a total of 107 compounds were considered for the evaluation of elicitations and grouped in compound families as phenols and mucic acid derivates, sterols, flavonoids, tannins, phenylpropanoid derivates, and terpenoids.

**Figure 3:**
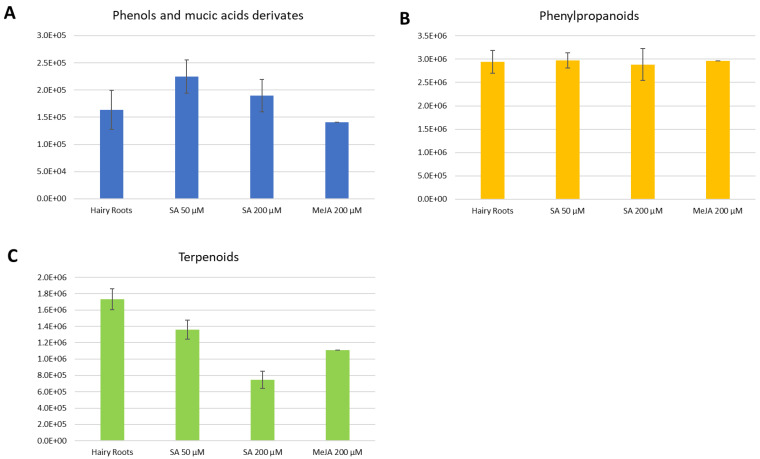
Average total intensities for each compound class present in each elicitation in the positive ionization mode. Error bars stand for the standard deviation of triplicates. (**A**). Phenols and mucic acids derivatives. (**B**). Phenylpropanoids. (**C**). Terpenoids.

**Figure 4:**
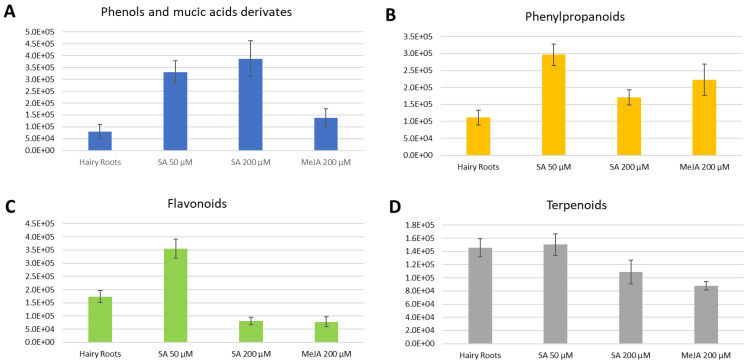
Average total intensities for each compound class present in each elicitation in the negative ionization mode. Error bars stand for the standard deviation of triplicates. (**A**). Phenols and mucic acids derivatives. (**B**). Phenylpropanoids. (**C**). Terpenoids. (**D**). Flavonoids.

Exogenous MeJA can alter the levels of various primary metabolites, including plant sugars, organic acids, and amino acids [11]. MeJA is involved in primary and secondary plant metabolism; moreover, it regulates plant bioactive compounds and enhances plants’ nutritional and medicinal value. It is also an important cellular regulator that controls plant development and defense responses to biotic and abiotic stresses [24,26]. Phytohormone induction prompts a response to adversity in plants and is the primary method for increasing the content of secondary metabolites in vegetables [27]. The application of MeJA promoted the accumulation of phenolic compounds, such as anthocyanin 3-galactoside, chlorogenic acid, and flavonols in apples [28]. In the present study, MeJA had a critical effect on individualized compounds, but overall, the production was similar to the hairy roots (Figure 3 and Figure 4).

SA is an endogenous signal substance that exists universally in plants and activates defense responses directly [29]. Moreover, the plant tissue culture technique per se elicits secondary metabolite production. Supplementation of SA to the culture medium or short-term exposure of the cultures to SA additively boosts the biosynthesis and accumulation of secondary metabolites [30]. In our study, SA managed to increase the production of phenols, phenylpropanoids, terpenoids, and flavonoids (Figure 3 and Figure 4); this is congruent with previous investigations where SA promotes the yields of secondary metabolites (including terpenoids) in medicinal plants and functions as one of the essential elicitors in hairy root cultures [29]. Due to its hormone-like activity, SA has been employed on different plant species in vivo and in vitro to explore its role in secondary metabolite synthesis and accumulation. These studies demonstrate that SA can efficiently recuperate the biosynthesis of secondary metabolites in plants, especially at 50 µM, but with individualized effects for 200 µM.

Considering the positive ionization measurements in a more detailed description, phenols and mucic acids were more present in the elicitations with SA, especially the concentration of 50 µM (Figure 3A).

For the phenylpropanoids, each elicitation’s tendency was unclear; they were expressed in both the control and the elicitations with SA and MeJA (Figure 3B). For terpenoids (Figure 3C), they were observed, as a general trend, to be more present in the control or hairy roots. However, this was followed by a considerable expression in the elicitation with SA of 50 µM.

On the other hand, clear tendencies were exhibited for the compounds observed in the negative ionization mode; for each compound family, changes were observed due to elicitation.

A significant increase of phenols and mucic acid derivates appears in the elicitations with SA, the main result being a greater expression of phenols in SA 200 µM (Figure 4A). The concentrations of phenylpropanoids also exceeded those in the control, this production being much more efficient in the presence of SA 50 µM (Figure 4B). However, MeJA also showed a considerable increase in the production of phenylpropanoids. The presence of flavonoids was visibly higher in the presence of the elicitor SA at 50 µM than in the other treatments (Figure 4D).

Similarly to the case in positive ionization measurement, a decrease in the concentration of terpenoids was also observed (Figure 4C).

### 2.2. Especific Effect in the Identified Compounds with MeJA and SA as Elicitors

The fold change from the normalized signals of compounds with the assignation of putative identifications was evaluated to compare each elicitation’s effect on their relative concentration. Higher observed fold changes are described in Table 1. The complete list of compounds can be consulted in Tables S5–S8 in the Supplementary Materials.

Compound intensities were evaluated using heat maps to visualize metabolite overexpression for each elicitation, and dendrograms grouped the compounds with similar behavior for the different applied treatments.

**Figure 5:**
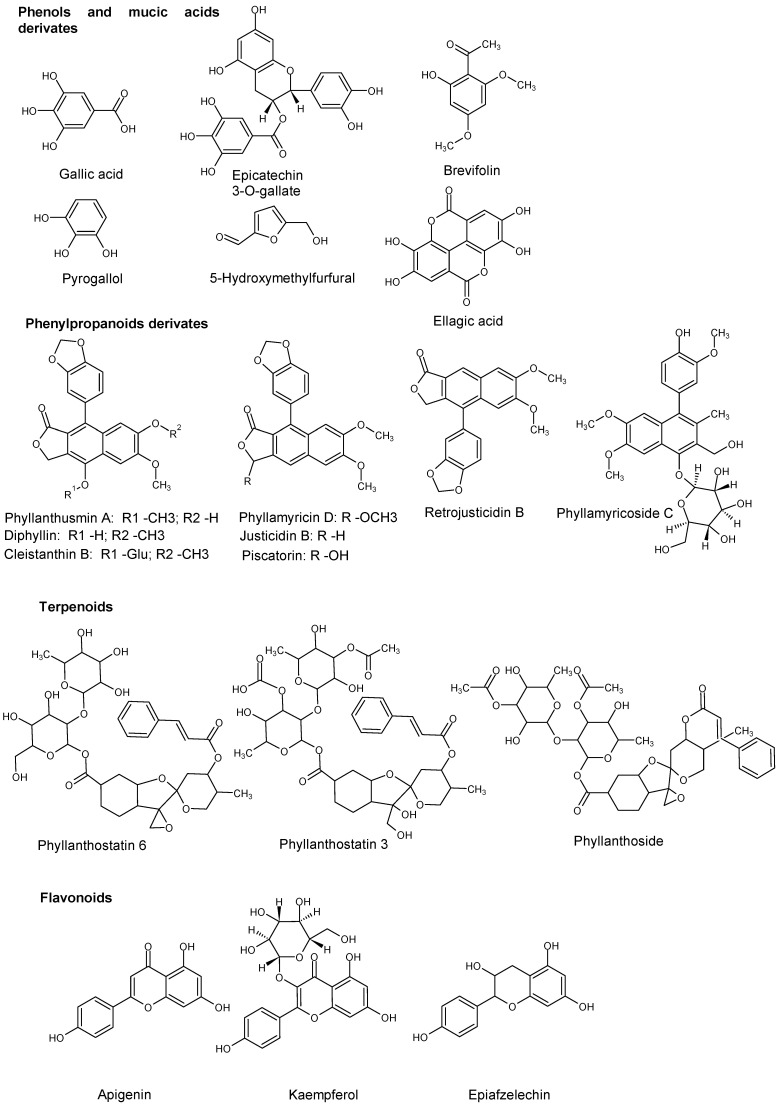
Structures of interesting compounds found in *Phyllanthus acuminatus* presented an increase in concentration in elicitation experiments.

The specific elicitation effect was also evaluated, considering compound classes. The molecular structures of listed interesting compounds are shown in Figure 5.

#### 2.2.1. Specific Phenols and Mucic Acids Derivates Evaluation

**Figure 6:**
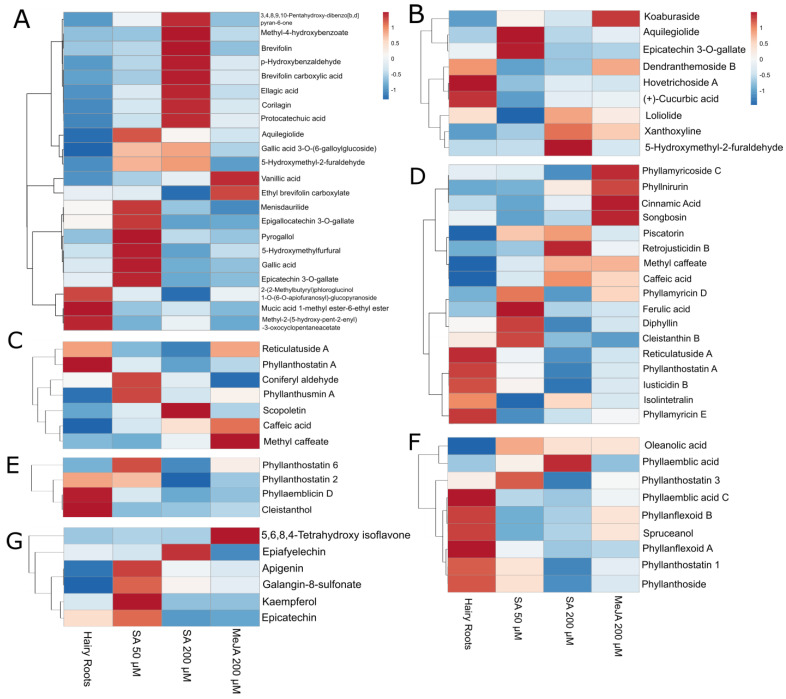
Specific compound variation with elicitation treatments, measurements in negative (**A**,**C**,**E**,**G**) and positive (**B**,**D**,**F**) ionization modes. Phenols and mucic acids derivates (**A**,**B**). Phenylpropanoids (**C**,**D**). Terpenoids (**E**,**F**). Flavonoids (**G**) Dendrograms grouped the compounds with similar behavior for the different applied treatments.

Aquilegiolide and epicatechin-3-O-gallate were elicited the most with SA 50 µM. Specific phenols and mucic acids such as gallic acid 3-O-(6-galloylglucoside), 5-hydroxymethyl-2-furaldehyde, menisdaurilide, epigallocatechin 3-O-gallate, pyrogallol, 5-hydroxymethylfurfural, and gallic acid were best elicited with SA 50 µM. Other phenols were elicited with SA 200 µM, e.g., 3,4,8,9,10-pentahydroxy-dibenzo[b,d] pyran-6-one, methyl-4-hydroxybenzoate, brevifolin, p-hydroxybenzaldehyde, brevifolin carboxylic acid, ellagic acid, corilagin, and protocatechuic acid stand out. Compounds such as vanillic acid, ethyl brevifolin carboxylate, and koaburaside were elicited in the presence of MeJA (Figure 6A,B).

The results shown are congruent with other studies where gallic acid, ellagic acid, 1β,6-di-*O*-galloylglucose, mucic acid 1,4-lactone methyl ester 5-*O*-gallate, and mucic acid dimethyl ester 2-*O*-gallate were found in other species such as *Phyllanthus emblica* and now are also shown to be present in *Phyllanthus acuminatus*. Also, corilagin and geraniin were identified in *Phyllanthus niruri* and *Phyllanthus muellerianus,* respectively [1].

Individualization for every compound should be highlighted. This means that an elicitor can be effective for a specific compound even if the elicitation did not occur for the entire phenol family. One promising compound elicited with SA 50 µM is gallic acid, which has an effect as an antiulcer, anti-jaundice, anti-inflammatory, and antioxidant and has been identified in other species such as *Phyllanthus emblica, Phyllanthus ninuri, Phyllanthus virgatus, Phyllanthus amarus*, and other members of the genus. SA had a positive effect eliciting Epicatechin 3-O-gallate, which has been proven to have antiviral activities in *Phyllanthus orbicularis*. Other bioactive compounds are pyrogallol and 5-Hydroxymethylfurfural with antitumor, anti-inflammatory, and antioxidant pharmacological effects, respectively. Brevifolin, which was far more present in the SA 200 µM elicitation, has proven to have a hepatoprotective effect in in vivo studies [24]. SA also influenced ellagic acid concentration, which has antioxidant, antidiabetic, and antitumor pharmacological effects [31].

#### 2.2.2. Specific Phenylpropanoids Evaluation

Among the elicited phenylpropanoids, the results varied greatly. Some compounds, such as reticulatuside A and phyllanthostatin A, were more present in hairy roots control (Figure 6C). Other compounds with bioactive properties, such as coniferyl aldehyde and phyllanthusmin A, were expressed more in the elicitation of SA 50 µM. At the same time, scopoletin was more present in the SA 200 µM elicitation (Figure 6C). MeJA had an eliciting effect on other phenylpropanoids, such as methyl caffeate and caffeic acid.

Also, a series of interesting compounds were elicited with SA 50 µM, such as phyllamyricin D, ferulic acid, diphyllin, and cleistanthin B (Figure 6D). Compounds such as piscatorin, methyl caffeate, and caffeic acid, in addition to retrojusticidin B, were also elicited with SA 200 µM.

The MeJA elicitor had positive results in compounds such as phyllamyricoside C, phyllnirurin, cinnamic acid, and songbosin. However, a series of phenylpropanoids, such as isolintetralin and phyllamyricin E, were more present in hairy roots (Figure 6D).

In the identifications of phenylpropanoids, there is no general tendency to favor the production of phenylpropanoids in the elicitation conditions. Nevertheless, some compounds have an effect that must be considered individually. Other papers have identified similar phenylpropanoids in other *Phyllanthus* species, such as justicidin, phyllanthostatin A, and cleistanthin in *P. brasiliensis* and the known antioxidant cinnamic acid in *Phyllanthus urinaria* [20]. Among the most interesting phenylpropanoids with an antitumor effect is phyllanthusmin A, which was favorably expressed in the elicitation of SA 50 µM [1].

A series of compounds elicited with SA 50 µM and 200 µM were found, such as phyllamyricin D and justicidin B, which show anti-inflammatory effects, and diphyllin, cleistanthin B, and piscatorin with antitumor effects [12]. Two other promising phenylpropanoids are retrojusticidin B and phyllamyricoside C, which have a proven anti-HIV pharmacological effect and can be elicited with SA and MeJA, respectively [32].

#### 2.2.3. Specific Terpenoids Evaluation

Bioactive compounds, such as phyllanthostatin 6, oleanolic acid, and phyllanthostatin 3 reached better production during the elicitation with SA 50 µM (Figure 6E). MeJA did not show promising results for eliciting most of the terpenoid family.

Well-known bioactive compounds, such as phyllanthostatin 3, oleanolic acid, and phyllanthoside, reached better production during the elicitation with SA 50 µM (Figure 6F). Furthermore, phyllaemblic acid was more present in SA 200 µM elicitation.

The results shown are congruent with other studies where spruceanol was found on *Phyllanthus acidus* [33], *P. urinaria*, *Phyllanthus songboiensis*, *P. oxyphyllus*, and *P. reticulatus* [32]; other compounds, such as oleanolic acid, were also reported on *P. urinaria* [22]. In addition, phyllanthoside, which was previously reported in *P. acuminatus* [1] and has an antitumor effect, showed no increment within the treatments.

#### 2.2.4. Specific Flavonoids Evaluation

In the group of flavonoids, many compounds showed more production with the tested elicitation conditions. Most flavonoids, such as apigenin, galangin-8-sulfonate, kaempferol, and (-)-epicatechin, were favored when elicited with SA 50 µM (Figure 6G). A compound elicited with SA 200 µM was (-)-epiafzelechin, and MeJA positively affected 5,6,8,4′-tetrahydroxy isoflavone production.

In the group of flavonoids, many compounds showed more production with the tested elicitation conditions. Most flavonoids were favored when elicited with SA 50 µM, such as apigenin, galangin-8-sulfonate, kaempferol, and epicatechin. Apigenin and kaempferol are effective antioxidants on *Phyllanthus emblica*, *Phyllanthus acidus*, *Phyllanthus ninuri*, and *Phyllanthus virgatus* [1]. A compound elicited with SA 200 µM was epiafzelechin, and MeJA positively affected 5,6,8,4′-tetrahydroxy isoflavone, as reported earlier, in *Phyllanthus atropurpureus* [1].

## 3. Materials and Methods

### 3.1. Hairy Root Elicitation

#### 3.1.1. Hairy Root Preparation and Cultivation Conditions

The in vitro plant material of *Phyllanthus acuminatus*, specifically hairy roots, was provided by Centro de Investigación en Biotecnología at Tecnológico de Costa Rica. The hairy roots were weighed using an analytical balance in a laminar flow chamber. For this, segments of roots without callus were cut, taken, and placed on filter paper to absorb and remove the excess culture medium. The hairy root segments were then placed on the balance until they reached a weight of 100 mg for each flask with a volume of 100 mL of 25% M&S culture medium.

The culture conditions for each elicitation test in 250 mL flasks were as in other studies: Photoperiod of 16 h light/8 h darkness, diffused light, and temperature of 25 ± 2 °C, with a culture time of four weeks [34]. The agitation for these experiments corresponded to 100 rpm.

For the hairy root elicitation process, three concentrations of elicitors were tested (200 μM of salicylic acid, 50 μM of salicylic acid, and 50 μM of methyl jasmonate [13,33,35]). The sample size for each elicitation assay in 250 mL flasks corresponded to n = 40 for each elicitor concentration.

#### 3.1.2. Elicitation Process on Hairy Roots

To carry out the elicitation, the elicitor corresponding to the trial was added at the beginning of the third week of the culture (day18). The flasks were placed in a laminar flow chamber, and the concentration corresponding to the elicitor was added to each (200 μM S.A, 50 μM S.A, and 50 μM of MeJA, respectively). After this, the flasks were again placed under agitation, as has been done in various studies [34,35]. Experiments were carried out in three biological replicates.

#### 3.1.3. Preparation of Elicited and Non-Elicited Root Sample

The plant material was subjected to continuous drying in a laboratory tray dryer in two phases, the first at room temperature for 48 h and the second at 50 °C for 2 h, finishing with grinding of the material.

### 3.2. Preparing the Extracts

For each treatment, three repetitions were created in microtubes of 0.15 g each of dried root material and 1.7 mL of absolute ethanol, and two glass microspheres were added to each tube. The resulting 15 tubes were added to a Retsch (Haan, Germany) automatic macerator for three series of 5 min durations at 24 hits per second. After the maceration, the extracts were left to rest for one hour at room temperature and later filtered with Whatman™ Puradisc 25 mm 0.2 µm filters. Extracts were evaporated at room temperature in a speed vacuum system RVC 2-18 Cdplus (Martin Christ, Osterode am Harz, Germany).

### 3.3. Determining Phytochemistry Profile Using High-Resolution Mass Spectrometry

Samples were reconstituted in 10% acetonitrile and then measured using a Xevo G2-XS quadrupole time-of-flight mass spectrometer coupled with an ACQUITY UPLC H-Class (Waters Corporation, Wilmslow, UK). A 1 µL sample injection was separated with a BEH-C18 column (2.1 mm × 100 mm, 1 µm) using a mobile phase gradient of A (water, 0.05% formic acid) and B (acetonitrile, 0.05% formic acid), and setting a flow of 0.5 mL/min. The gradient consisted of maintaining 5% B for 5 min, then increasing to 80% at 10 min and, subsequently, to 100% at 11 min and holding until 13 min, finishing by equilibrating the column to initial conditions. The column temperature was set to 50 °C. The mass spectrometer was configured to use a capillary voltage of 2 kV, a 40 V sampling cone, and a source offset of 80 V. Source temperatures were set at 125 °C and 600 °C for the desolvation temperature, and gas flows were set to 150 L/h for the cone gas and 1000 L/h for the desolvation gas. Measurements were made using the MS^E^ independent data acquisition mode in positive and negative polarities in high-resolution mode with a mass range from 50–1000 *m*/*z*, scan time of 0.25 s, and collision energy ramp from 20 V to 40 V for the high energy function.

Mass spectra were analyzed using Progenesis Q.I. (Waters Corporation, UK) software with Progenesis MetaScope for compound identification, using an in-house structure data file (SDF) comprising a database of 512 compounds containing previously reported structures in this genus [1,5] sorted by compound type in families as phenols and mucic acids, sterols, flavonoids, tannins, phenylpropanoids, and terpenoids. Theoretical fragmentation matching was achieved by Progenesis Metascope algorithms, setting precursors and fragment tolerance to 10 ppm. Putative identification of compounds was performed by accepting identification of structures with a mass error lower than 5 ppm, a match with the theoretical isotopic distribution better than 90%, and assigning at least three fragments from the theoretical fragmentation of the molecules with mass accuracy better than 10 ppm.

Progenesis Q.I was used to normalize peak area data using the “normalize to all compounds” function for the relative comparison. A summary of normalized areas by compound type was used for their relative concentrations.

Heat maps for specific compounds were calculated using specific normalized areas and generated correlating rows average with ClustVis [36].

Fold changes were calculated as relative changes (A_Treatment_ − A_Control_/A_Control_) to visualize the relative concentration decrease where A corresponds to the average normalized area of each compound.

## 4. Conclusions

High-resolution mass spectrometry has proven to be an outstanding tool for evaluating plant extracts due to the highly informative content of data-independent acquisition measurements. The use of non-targeted approaches allowed the relative evaluation of the effectiveness of elicitors in *Phyllanthus acuminatus* hairy root cultures. SA 50 μM produced the highest elicitation in phenols, music acid derivates, and flavonoids, increasing their relative abundance by more than 100% relative to control and 300% for phenylpropanoid derivates. However, any treatment increased the concentration of terpenoids besides phyllanthostatin 6, which has been found to have antiviral, anticancer, and antibacterial properties, making it a promising candidate for drug development.

## Figures and Tables

**Table 1:** Compounds with higher fold change in normalized signals due to elicitation.

	Fold Change		
Compound	SA 50 µM	SA 200 µM	MeJA 200 µM
**Phenols and mucic acid derivates**			
5-Hydroxymethyl-2-furaldehyde	1100	1186	73
Protocatechuic acid	4.5	16.4	6.0
3,4,8,9,10-Pentahydroxy-dibenzo[b,d] Pyran-6-one	40.4	106.7	11.2
Ellagic acid	53.6	178.4	40.4
Corilagin	45.5	152.0	46.1
Pyrogallol	153.8	13.0	2.2
Aquilegiolide	29.1	16.3	10.4
Brevifolin carboxylic acid	382.8	2822.3	756.8
Gallic acid	2.7	−0.8	−0.6
Epicatechin 3-O-gallate	2.4	−1.0	−0.8
Epigallocatechin 3-O-gallate	1.2	−1.0	−0.9
Xanthoxyline	0.9	5.7	4.4
p-hydroxybenzaldehyde	1.2	6.6	1.8
Koaburaside	2.3	1.1	4.7
**Phenylpropanoids**			
Caffeic acid	2.9	5.1	7.1
Scopoletin	43.1	160.8	24.5
Phyllanthusmin A	2.3	0.8	1.3
Piscatorin	2.5	2.9	1.3
Phyllamyricin D	5.0	−0.4	3.7
Ferulic acid	9.7	0.4	1.1
**Terpenoids**			
Phyllanthostatin 6	2.0	−0.3	1.0
**Flavonoids**			
Kaempferol	4.7	−1.0	−1.0
Apigenin	6.5	3.0	2.4
Galangin-8-sulfonate	7.0	4.1	3.2

## Data Availability

Data are contained within the article and Supplementary Materials.

## Supplementary Materials

The following supporting information can be downloaded at https://www.mdpi.com/article/10.3390/molecules29010080/s1. Tables S1–S4: Descriptors for putative identification assignment. Tables S5–S8: Fold changes for all the identified compounds. Table S9. Compounds with higher fold change in normalized signals due to elicitation.
