# Supplementary material for: Evaluation of Salicylic Acid and Methyl Jasmonate as Elicitors in Phyllanthus acuminatus Hairy Roots by Non-Targeted Analysis Using High-Resolution Mass Spectrometry
Source: Molecules. 2023 Dec 22;29(1):80. doi: 10.3390/molecules29010080 (PMC10780090; doi:10.3390/molecules29010080)
Supplement: Supplementary file 1 [file molecules-29-00080-s001.zip › molecules-2714731-supplementary.pdf]

Table S1. Description of putative identification assignation for phenols and mucic acid derivates.

Compound	Formula	Retention time (min)	Mass error (ppm)	Adducts	Precursor ion (m/z)	Assigned fragments (m/z)
Mucic acid 1-methyl ester-6-ethyl ester	C9H16O8	0.6	-3.7	M-H	251.0763	179, 101
Brevifolin	C12H8O6	0.9	-0.6	M-H	247.0247	233, 205, 89, 125
5-Hydroxymethyl-2-furaldehyde	C6H6O3	3.5	-4.4	M-H	125.0239	108, 79
			9.1	M+H	127.0401	127, 111,69
Protocatechuic acid	C7H6O4	3.5	-4.3	M-H	153.0187	121, 108
p-Hydroxybenzaldehyde	C7H6O2	3.9	-4.6	M-H	121.0289	92
1,6-O-galloyl-glucose	C20H20O14	4.1	-1.3	M-H	483.0774	313, 271,211
Vanillic acid	C8H8O4	4.3	-4.1	M-H	167.0343	152, 135, 123
Methyl-4-hydroxybenzoate	C8H8O3	6.2	-4.6	M-H	151.0394	136, 121
Ethyl brevifolin carboxylate	C15H12O8	6.6	1.4	M-H	319.0464	287, 151, 125
Menisdaurilide	C8H8O3	6.7	-2.3	M-H	151.0397	137,125, 109
3,4,8,9,10-Pentahydroxy-dibenzo[b,d] pyran-6-one	C13H8O7	6.8	-1.1	M-H	275.0194	247, 169, 125
Ellagic acid	C14H6O8	6.8	-2.1	M-H	300.9984	275, 259
Corilagin	C27H22O18	6.8	-0.8	M-H	633.0729	575, 463,300
Pyrogallol	C6H6O3	7.0	-1.8	M-H	125.0242	109, 92
Aquilegiolide	C8H8O3	7.0	-0.4	M-H	151.0400	137,125, 109
			7.8	M+H-H_2O	135.0452	135, 105, 92
Brevifolin carboxylic acid	C13H8O8	7.0	-2.5	M-H	291.0139	247, 217, 125
5-Hydroxymethylfurfural	C6H6O3	7.4	-2.7	M-H	125.0241	109, 97
Gallic acid	C7H6O5	7.4	-2.3	M-H	169.0139	137, 125
Epicatechin 3-O-gallate	C22H18O10	7.4	-1.7	M-H	441.0820	407, 331, 289, 169, 125
			2.3	M+H	443.0983	409, 273, 153, 123
Epigallocatechin 3-O-gallate	C23H20O10	7.7	0.2	M-H	455.0985	305, 137
2-(2-Methylbutyryl)phloroglucinol 1-O-(6-O--apiofuranosyl)-glucopyranoside	C22H32O13	7.8	1.2	M-H	503.1776	323, 279, 247, 179
Methyl-2-(5-hydroxy-pent-2-enyl)-3-oxocyclopentaneacetate	C14H22O4	8.9	-3.3	M-H	253.1437	235, 211
Xanthoxyline	C10H12O4	5.0	3.5	M+H	197.0815	179, 163, 149, 137
p-hydroxybenzaldehyde	C7H6O2	7.4	2.5	M+H	123.0444	106, 89
Loliolide	C11H16O3	7.6	-1.8	M+H	197.1169	163, 139, 123
Hovetrichoside A	C23H30O11	7.6	-0.7	M+H	483.1857	469, 321, 167
Cucurbic acid	C12H20O3	8.5	8.4	M+Na	235.1322	217, 156
Koaburaside	C14H20O9	9.4	-8.7	M+H	315.1045	315, 297
Dendranthemoside B	C19H32O8	9.9	0.3	M+Na	411.1991	293, 226, 177

Table S2. Description of putative identification assignation for Phenylpropanoids

Compound	Formula	Retention time (min)	Mass error (ppm)	Adducts	Precursor ion (m/z)	Assigned fragments (m/z)
Caffeic acid	C9H8O4	4.4	-4.5	M-H	179.0342	145, 133, 123,108
			7.6	M+H-H2O	163.0403	153, 125, 107
Coniferyl aldehyde	C10H10O3	6.7	-3.6	M-H	177.0551	161, 145, 135, 123
Scopoletin	C10H8O4	6.7	-2.2	M-H	191.0346	180, 173, 145, 117
Methyl caffeate	C10H10O4	7.4	-3.5	M-H	193.0499	161, 145, 15
Reticulatuside A	C32H34O16	8.2	4.3	M-H	673.1803	465, 379, 263, 221
Phyllanthostatin A	C29H30O13	8.6	1.0	M-H	585.1619	451, 423, 379, 121
			0.4	M+Na	609.1581	447, 187
Phyllanthusmin A	C21H16O7	9.7	-0.5	M-H	379.0821	364, 334, 304
			2.8	M+H	381.0980	363, 333, 305
Piscatorin	C21H16O7	9.7	2.9	M+H-H2O	363.0874	333, 305, 289
Phyllamyricin E	C22H18O7	9.7	3.3	M+H, M+Na	395.1138	381, 363, 333, 305
Retrojusticidin B	C21H16O6	9.5	2.7	M+H-H2O	347.0924	355, 321, 307, 289
Phyllnirurin	C20H22O5	9.4	4.3	M+H-H2O	325.1449	313, 295, 283, 135
Cinnamic Acid	C9H8O2	9.3	8.1	M+H	149.0609	147, 115, 105, 91
Phyllamyricin D	C23H20O8	9.2	1.6	M+Na	447.1057	387, 365, 335
Iusticidin B	C21H16O6	8.6	5.0	M+H-H2O, M+H, M+Na	365.1038	365, 347, 335, 321
Phyllamyricoside C	C27H32O11	8.6	-3.8	M+H-H2O	515.1891	451, 443
Songbosin	C20H20O6	8.5	5.2	M+H-H2O	339.1245	325, 311, 177, 137
Reticulatuside A	C32H34O16	8.2	0.1	M+H, M+Na	675.1920	697, 543, 3812, 333, 305
Cleistanthin B	C27H26O12	8.2	1.5	M+H	543.1505	403, 381, 363
Diphyllin	C21H16O7	8.2	2.7	M+H-H2O, M+H	381.0979	363,351, 337, 333, 305
Methyl caffeate	C10H10O4	7.4	5.7	M+H-H2O	177.0557	175, 163, 145, 123
Ferulic acid	C10H10O4	7.2	5.9	M+H-H2O	177.0558	163, 147, 123
Coniferyl aldehyde	C10H10O3	7.2	5.1	M+H	179.0712	163, 145, 135, 105
Isolintetralin	C23H28O6	10.6	3.5	M+H-H2O	383.1867	368, 352, 337, 285, 133

Table S3. Description of putative identification assignation for terpenoids.

Compound	Formula	Retention time (min)	Mass error (ppm)	Adducts	Precursor ion (m/z)	Assigned fragments (m/z)
Phyllemblicin D	C21H34O13	3.2	-1.5	M-H	493.1919	445, 197, 150
Phyllaemblic acid C	C15H24O8	3.5	-2.9	M-H	331.1389	
Phyllanthostatin 6	C36H48O16	8.7	0.4	M-H	735.2872	615, 469, 427, 247163
				M+Na	759.2826	469, 451, 331, 245
Phyllanthostatin 2	C40H52O18	9.3	-0.5	M-H	819.3077	789, 747, 703, 661, 601, 455
Phyllanthoside	C40H52O17	10.1	3.2	M-H	803.3157	
			0.1	M+H	805.3278	429, 377, 189
Cleistanthol	C20H28O3	10.8	-3.0	M-H	315.1956	297, 239, 183
Phyllaemblic acid C	C15H24O8	8.3	1.4	M+H-H$_2$O	315.1443	299, 281, 233, 156
Phyllanthostatin 3	C40H54O18	9.2	-3.6	M+Na	845.3173	831, 339
Phyllanflexoid A	C20H26O3	9.6	4.3	M+H-H$_2$O, M+H	297.1862	297, 279, 211, 199
Phyllanflexoid B	C20H24O3	9.7	1.6	M+H	313.1803	295, 277, 223, 178
Phyllanthoside	C40H52O17	9.8	-2.3	M+H, M+Na	827.3078	679, 636, 339
Phyllanthostatin 1	C40H52O17	10.1	-2.2	M+Na	827.3079	451, 399, 189
Spruceanol	C20H28O2	10.2	-0.9	M+H-H$_2$O	283.2054	267, 227, 213, 199
Oleanolic acid	C30H48O3	11.3	-0.3	M+H-H$_2$O	439.3569	393, 269, 203, 189

Table S4. Description of putative identification assignation for flavonoids.

Compound	Formula	Retention time (min)	Mass error (ppm)	Adducts	Precursor ion (m/z)	Assigned fragments (m/z)
Kaempferol	C15H10O6	6.7	-2.2	M-H	285.0398	269, 255, 161, 137, 125
Epicatechin	C15H14O6	6.7	-2.5	M-H	289.0710	259, 229, 179, 151, 125
Epiafzelechin	C15H14O5	7.1	9.3	M-H	273.0794	259, 245, 151, 123
5,6,8,4-Tetrahydroxy isoflavone	C15H10O6	7.5	-1.7	M-H	285.0400	273, 163, 151
Apigenin	C15H10O5	8.7	-2.1	M-H	269.0450	239, 211, 161, 135
Galangin-8-sulfonate	C15H10O8S	8.7	-1.5	M-H	349.0018	269, 161, 117

Table S5. Fold change in normalized signals for phenols and mucic acid derivates due to elicitation.

Compound	Fold Change		
	SA 50 μM	SA 200 μM	MeJA 200 μM
Mucic acid 1-methyl ester-6-ethyl ester	-0.8	-0.7	-0.9
Brevifolin	0.3	7.1	-0.2
5-Hydroxymethyl-2-furaldehyde	1100.2	1186.2	73.0
Protocatechuic acid	4.5	16.4	6.0
p-Hydroxybenzaldehyde	1.2	6.6	1.8
1,6-O-galloyl-glucose			
Vanillic acid	1.0	1.9	5.3
Methyl-4-hydroxybenzoate	0.1	4.4	0.4
Ethyl brevifolin carboxylate	0.0	-0.2	0.2
Menisdaurilide	1.1	-0.6	-1.0
3,4,8,9,10-Pentahydroxy-dibenzo[b,d] pyran-6-one	40.4	106.7	11.2
Ellagic acid	53.6	178.4	40.4
Corilagin	45.5	152.0	46.1
Pyrogallol	153.8	13.0	2.2
Aquilegiolide	29.1	16.3	10.4
Brevifolin carboxylic acid	382.8	2822.3	756.8
5-Hydroxymethylfurfural	2.9	-0.8	-0.1
Gallic acid	2.7	-0.8	-0.6
Epicatechin 3-O-gallate	2.4	-1.0	-0.8
Epigallocatechin 3-O-gallate	1.2	-1.0	-0.9
2-(2-Methylbutyryl)phloroglucinol 1-O-(6-O--apiofuranosyl)-glucopyranoside	-0.6	-1.0	-0.5
Methyl-2-(5-hydroxy-pent-2-enyl)-3-oxocyclopentaneacetate	-0.8	-0.5	-0.8
Xanthoxyline	0.9	5.7	4.4
p-hydroxybenzaldehyde	1.2	6.6	1.8
Loliolide	-0.4	0.1	0.0
Hovetrichoside A	-0.8	-0.7	-0.7
Cucurbic acid	-0.2	-0.1	-0.1
Koaburaside	2.3	1.1	4.7
Dendranthemoside B	-0.4	-0.4	0.0

Table S6. Fold change in normalized signals for phenylpropanoids due to elicitation.

Compound	Fold Change		
	SA 50 μM	SA 200 μM	MeJA 200 μM
Caffeic acid	2.9	5.1	7.1
Coniferyl aldehyde	0.8	-0.2	-0.9
Scopoletin	43.1	160.8	24.5
Methyl caffeate	-0.1	0.5	1.6
Reticulatuside A	-0.4	-0.5	0.0
Phyllanthostatin A	-0.7	-1.0	-0.8
Phyllanthusmin A	2.3	0.8	1.3
Piscatorin	2.5	2.9	1.3
Phyllamyricin E	-0.7	-0.5	-0.4
Retrojusticidin B	0.1	1.2	0.4
Phyllnirurin	0.1	2.9	5.6
Cinnamic Acid	-0.2	0.2	1.1
Phyllamyricin D	5.0	-0.4	3.7
Iusticidin B	-0.4	-0.9	-0.5
Phyllamyricoside C	0.0	-0.2	0.4
Songbosin	-0.5	-0.3	0.9
Reticulatuside A	-0.1	-0.2	-0.2
Cleistanthin B	0.1	-0.1	-0.1
Diphyllin	0.0	0.0	0.0
Methyl caffeate	0.5	1.0	1.0
Ferulic acid	9.7	0.4	1.1
Coniferyl aldehyde	0.8	-0.2	-0.9
Isolintetralin	-0.7	-0.1	-0.4

Table S7. Fold change in normalized signals for terpenoids due to elicitation.

Compound	Fold Change		
	SA 50 μM	SA 200 μM	MeJA 200 μM
Phyllemblicin D	-0.6	-0.8	-0.7
Phyllaemblic acid C	-0.3	-0.3	-0.2
Phyllanthostatin 6	2.0	-0.3	1.0
Phyllanthostatin 2	-0.1	-0.8	-0.6
Phyllanthoside	-0.3	-0.8	-0.6
Cleistanthol	-1.0	-1.0	-0.9
Phyllaemblic acid C	-0.3	-0.3	-0.2
Phyllanthostatin 3	0.4	-0.6	-0.1
Phyllanflexoid A	-0.6	-0.9	-0.8
Phyllanflexoid B	-0.6	-0.6	-0.3
Phyllanthoside	-0.3	-0.8	-0.6
Phyllanthostatin 1	-0.3	-0.9	-0.5
Spruceanol	-0.4	-0.3	-0.2
Oleanolic acid	0.5	0.4	0.4

Table S8. Fold change in normalized signals for flavonoids due to elicitation.

Compound	Fold Change		
	SA 50 μM	SA 200 μM	MeJA 200 μM
Kaempferol	4.7	-1.0	-1.0
Epicatechin	0.5	-1.0	-1.0
Epiafzelechin	0.0	0.4	-0.2
5,6,8,4-Tetrahydroxy isoflavone	0.5	0.3	9.7
Apigenin	6.5	3.0	2.4
Galangin-8-sulfonate	7.0	4.1	3.2

Table S9. Compounds with higher fold change in normalized signals due to elicitation.

Compound	Fold Change		
	SA 50 μM	SA 200 μM	MeJA 200 μM
**Phenols and mucic acid derivates**			
5-Hydroxymethyl-2-furaldehyde	1100	1186	73
Protocatechuic acid	4.5	16.4	6.0
3,4,8,9,10-Pentahydroxy-dibenzo[b,d] Pyran-6-one	40.4	106.7	11.2
Ellagic acid	53.6	178.4	40.4
Corilagin	45.5	152.0	46.1
Pyrogallol	153.8	13.0	2.2
Aquilegiolide	29.1	16.3	10.4
Brevifolin carboxylic acid	382.8	2822.3	756.8
Gallic acid	2.7	-0.8	-0.6
Epicatechin 3-O-gallate	2.4	-1.0	-0.8
Epigallocatechin 3-O-gallate	1.2	-1.0	-0.9
Xanthoxyline	0.9	5.7	4.4
p-hydroxybenzaldehyde	1.2	6.6	1.8
Koaburaside	2.3	1.1	4.7
**Phenylpropanoids**			
Caffeic acid	2.9	5.1	7.1
Scopoletin	43.1	160.8	24.5
Phyllanthusmin A	2.3	0.8	1.3
Piscatorin	2.5	2.9	1.3
Phyllamyricin D	5.0	-0.4	3.7
Ferulic acid	9.7	0.4	1.1
**Terpenoids**			
Phyllanthostatin 6	2.0	-0.3	1.0
**Flavonoids**			
Kaempferol	4.7	-1.0	-1.0
Apigenin	6.5	3.0	2.4
Galangin-8-sulfonate	7.0	4.1	3.2
